# Human neurocysticercosis unexpectedly caused by *Taenia martis* in Italy: a case report and literature review

**DOI:** 10.1186/s13071-026-07420-2

**Published:** 2026-05-15

**Authors:** Azzurra Santoro, Antonio Di Grazia, Marina Cuntrò, Elena Gervasi, Luca Baldassarri, Marco Arosio, Matteo Lacavalla, Simona Cherchi, Alessandra Ludovisi, Federica Santolamazza, Irene Tartarelli, Francesco Celani, Annibale Raglio, Edoardo Carretto, Adriano Casulli

**Affiliations:** 1 European Union Reference Laboratory for Parasites (EURL-P), Department of Infectious Diseases, Istituto Superiore Di Sanità, Viale Regina Elena 299, 00161 Rome, Italy; 2 European Union Reference Laboratory for Public Health On Helminths and Protozoa (EURL-PH-HP), Department of Infectious Diseases, Istituto Superiore Di Sanità, Rome, Italy; 3 WHO Collaborating Centre for the Epidemiology, Detection and Control of Cystic and Alveolar Echinococcosis (One Health), Department of Infectious Diseases, Istituto Superiore Di Sanità, Rome, Italy; 4 SC Clinical Microbiology and Virology, ASST “Papa Giovanni XXIII”, Bergamo, Italy; 5 Infectious Diseases Unit, ASST “Papa Giovanni XXIII”, Bergamo, Italy

**Keywords:** Neurocysticercosis, *Taenia martis*, Zoonosis, One Health

## Abstract

**Background:**

Cysticercosis is caused by the larval stages of *Taenia* and *Versteria* species. While *Taenia solium*, associated with a domestic life cycle, is the primary etiological agent of human (neuro)cysticercosis worldwide, sporadic cases involving *Taenia* species linked to wildlife have also been reported. A 72‑year‑old immunocompetent woman presented in October 2024 to the Emergency Department with paresthesia, gait imbalance, and severe headache. Neuroimaging revealed a <1‑cm edematous lesion in the frontal cortex, suspicious for metastasis. Surgical removal was performed. Histopathological examination showed necrotic tissue with abundant inflammatory infiltrate, ruling out neoplasia and raising suspicion of an infectious etiology. She was discharged in February 2025 with improved symptoms. Ongoing follow-up demonstrated progressive improvement leading to complete remission.

**Methods:**

Serological tests, coproparasitological examinations, and panfungal PCR targeting the 18S rRNA gene were performed. Intraoperative material, including formalin-fixed paraffin-embedded (FFPE) tissue samples, was collected and processed for histological, immunohistochemical, and molecular analyses. Histological sections were stained with hematoxylin and eosin (H&E) and Periodic acid–Schiff (PAS). Immunohistochemical analyses were carried out on FFPE sections to investigate infection with *Echinococcus* spp. For molecular analysis, genomic DNA was extracted from FFPE tissue using a dedicated commercial kit, following the manufacturer’s instructions. Mitochondrial gene targets (*cox1, nad1,* and 12S rRNA) were amplified by PCR and subsequently sequenced.

**Results:**

Serological tests, coproparasitological examinations, and panfungal PCR targeting the 18S rRNA gene were negative. Histological analysis showed an inflammatory lesion with a necrotic core, while immunohistochemical analyses were negative for *Echinococcus* spp. Molecular analysis was positive, and sequence and phylogenetic analyses identified the cestode *Taenia martis*.

**Conclusions:**

This finding underscores the need for clinicians to consider less common *Taenia* species as potential causes of cysticercosis and neurocysticercosis, particularly when serological tests are negative or inconclusive despite a clinically suggestive presentation.

**Graphical Abstract:**

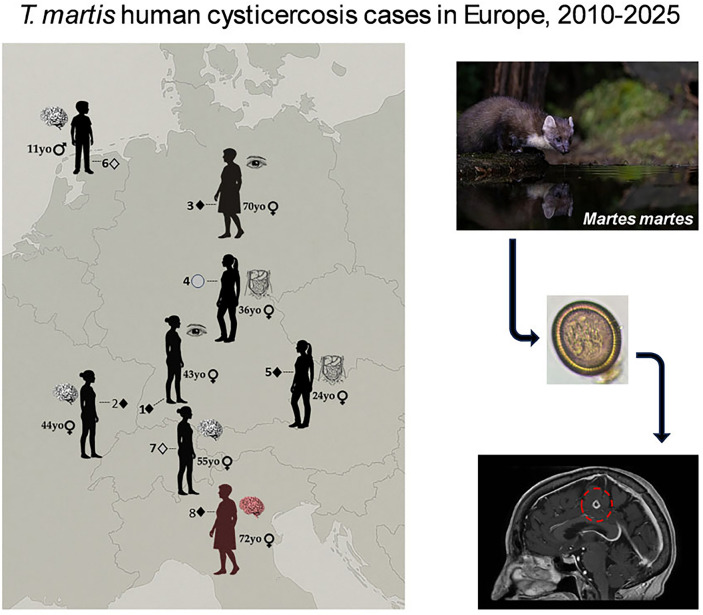

**Supplementary Information:**

The online version contains supplementary material available at 10.1186/s13071-026-07420-2.

## Background

Cysticercosis is a parasitic disease caused by the larval stage (metacestode) of several tapeworms of the genera *Taenia* and *Versteria*. It occurs when intermediate or dead-end hosts ingest their eggs, leading to the development of cysts in various sites, including organs, body cavities, or the subcutaneous tissue.

Humans are the definitive hosts of the tapeworms *Taenia solium*, *Taenia asiatica*, and *Taenia saginata*, whose larval stages, Cysticercus cellulosae, Cysticercus viscerotropica, and Cysticercus bovis, typically infect pigs and cattle. Human taeniasis occurs by ingestion of raw or undercooked meat from intermediate hosts carrying metacestodes, while human cysticercosis results from ingestion of eggs, with humans acting as accidental (dead-end) intermediate hosts [1]. Clinical signs depend on the number and location of cysticerci: in most tissues (muscles, skin, subcutaneous tissues, lungs, liver) they often remain asymptomatic and degenerate spontaneously. Central nervous system involvement causes neurocysticercosis (NCC), a serious condition with neurological and epileptic manifestations. NCC is classified as parenchymal or extra-parenchymal depending on cyst location.

*Taenia solium* is the leading cause of human cysticercosis and NCC worldwide, especially in India, South America, Southeast Asia, and sub-Saharan Africa [2]. Consequently, immunological tests detecting *T. solium* antibodies or antigens are widely available, although they do not perform at the same level [3]. However, molecular diagnostics have revealed rare cases of wildlife-transmitted cysticercosis in Europe, mainly due to *Taenia crassiceps* [1, 4] and *Taenia martis* [5–11], affecting both immunocompetent and immunocompromised individuals.

*Taenia martis* (Zeder, 1803) (Cestoda, Cyclophyllidea) is a zoonotic tapeworm of the Northern Hemisphere. Adults parasitize mustelids, especially the stone marten (*Martes foina*) and pine marten (*Martes martes*), and occasionally red foxes [12], dogs [13], and wild cats [1, 14]. Rodents ingest infective eggs and develop larval stages in the peritoneal or pleural cavities; predation completes the life cycle. Humans and nonhuman primates may become accidental intermediate hosts. To date, seven human cases of *T. martis* cysticercosis have been reported in Europe, involving cerebral, ocular, and peritoneal localizations [5–11].

To our knowledge, we report the first Italian case of neurocysticercosis caused by *T. martis*, providing also a brief overview of its epidemiological and clinical characteristics.

## The case

A 72-year-old woman presented in October 2024 with new-onset headache and left-sided weakness involving the arm and foot. Her medical history included chronic headaches without other comorbidities or chronic medications. A brain computed tomography (CT) scan showed a space-occupying right frontal lesion with surrounding edema, suspicious for metastasis. Routine blood tests were normal. Extensive diagnostic workup, including whole-body CT, positron emission tomography (PET)–CT, upper endoscopy, and colonoscopy, did not identify any primary neoplastic lesions.

**Fig. 1:**
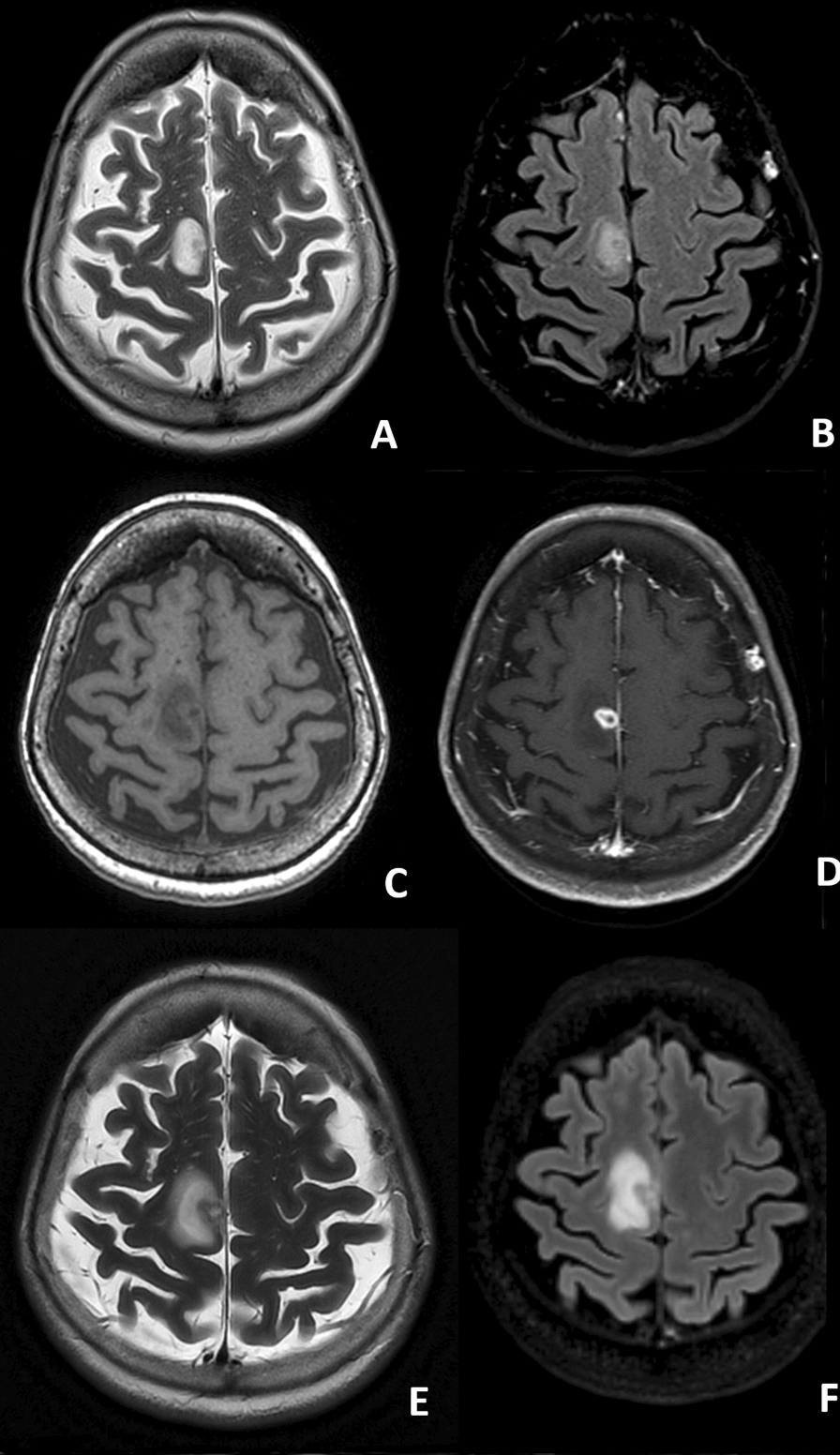
Diagnostic imaging in MRI: **A** axial T2‑weighted turbo spin echo (TSE) and **B** axial fluid-attenuated inversion recovery (FLAIR) images showing a hyperintense lesion with well‑defined margins and no evidence of perilesional edema. **C**–**E** Images acquired after steroid therapy: **C** axial T1-weighted image showing a mild increase in the size of the expansile lesion, with more indistinct margins; **D** post-contrast image demonstrating the appearance of a new nodular component within the lesion, exhibiting contrast enhancement; **E** axial T2‑weighted and **B** axial FLAIR images documenting the appearance of subtle perilesional edema

Brain magnetic resonance imaging (MRI) showed a single, small, ring-enhancing subcortical lesion (<1 cm) anterior to the right paracentral lobule (Fig. 1). Based on imaging findings, the patient was referred to the Neurosurgery Department at Papa Giovanni XXIII Hospital in Bergamo (Lombardy region, Italy) and underwent radiological follow-up. As the lesion persisted, she was admitted in mid-January 2025 for surgical resection. The procedure was uneventful. Histology excluded malignancy and showed necrotic, abscess-like material with abundant lymphoplasmacytic infiltration.

Suspecting an infectious etiology, the patient was transferred to the Infectious Diseases Unit for further diagnostic workup. She remained afebrile, although headache and mild left arm weakness persisted.

Serological tests for *Toxoplasma gondii*, human immunodeficiency virus (HIV), *Echinococcus* spp., and *Entamoeba histolytica* were negative. Serum fungal antigens (β-d-glucan, galactomannan) were also negative. Coproparasitological examinations were negative. The complete blood count showed no eosinophilia. A “panfungal” PCR (18S rRNA) performed on a stored intraoperative sample was negative.

Lumbar puncture yielded clear cerebrospinal fluid (CSF) with a normal opening pressure. CSF analysis was normal: white blood cell (WBC) 1 cell/mm^3^, protein 21 mg/dL, glucose 58 mg/dL; cytology showed very sparse lymphocytes. PCRs for *T. gondii* and *Mycobacterium tuberculosis* complex, cryptococcal antigen, and bacterial and fungal cultures were all negative, as were microscopy for acid-fast bacilli and mycobacterial culture.

The patient was discharged in late February 2025 with scheduled outpatient follow-up. A brain MRI performed before discharge showed no recurrence and reduced edema. In particular, the previous ring enhancement had disappeared, and a postoperative hypointense area was visible at the site of the exeresis. No pathological contrast enhancement was observed after contrast administration. At follow-up in March 2025, the patient reported slight improvement (persistent paresthesia in the lower limbs and weakness in the left hand). Laboratory tests remained within normal limits. Continued clinical and radiological follow-up was scheduled.

The serum and the CSF, as well as the formalin-fixed paraffin-embedded tissue (FFPE) sample, were submitted to the European Union Reference Laboratory for Parasites (EURL-P) at the Istituto Superiore di Sanità (Rome, Italy), for diagnostic testing aimed at assessing possible *Echinococcus* spp. or *Taenia* spp. involvement.

Both the serum and the CSF samples were tested using the LDBIO IgG western blot assay (LDBIO Diagnostics) and were negative for *Taenia solium*-specific antibodies.

**Fig. 2:**
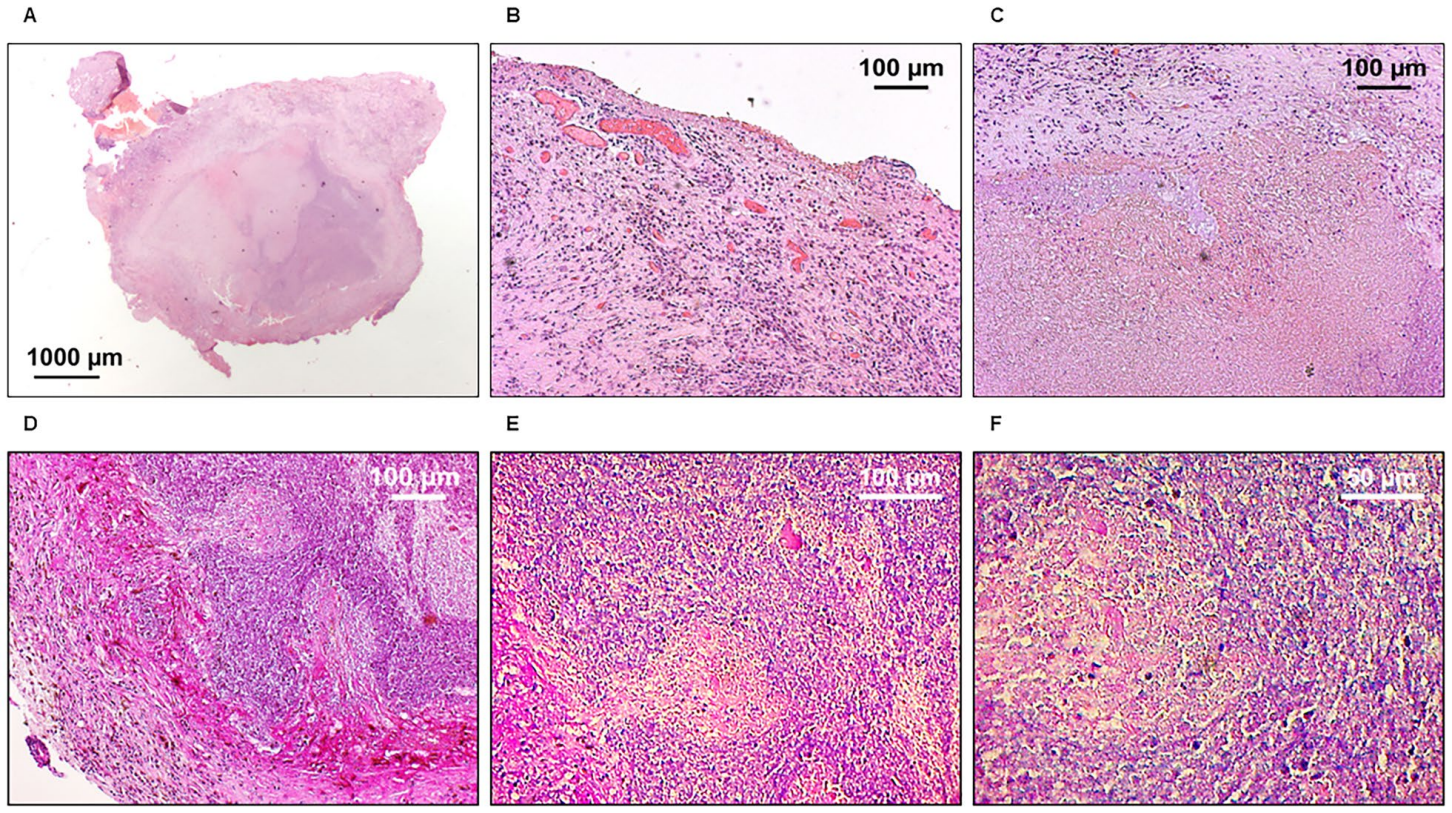
Representative H&E (**A**–**C**) and PAS staining (**D**–**F**) of paraffin-embedded (FFPE) sections of biopsy samples. The H&E staining shows brain tissue with inflammation plus red blood cells (**B**), and a necrotic area with undefined structures (**C**). A strongly PAS-positive necrotic area is visible (**D**), along with less PAS-intense cylindrical structures possibly representing elements of the larval stage of *T. martis* (**E**, **F**). Scale bars: 1000 µM (**A**), 100 µM (**B**–**E**), 50 µM (**F**)

Histological sections stained with hematoxylin and eosin (H&E) and Periodic acid–Schiff (PAS) revealed a granulomatous lesion with a central necrotic core (Fig. 2). Externally, the lesion displayed a delimiting zone rich in blood vessels and inflammatory cells, while within the central necrotic core, undefined structures were visible. No giant cells or eosinophils were observed. A strongly PAS-positive rim surrounded cylindrical structures measuring approximately 10–15 µm.

In addition, to exclude possible infections by *Echinococcus granulosus* or *Echinococcus multilocularis*, immunohistochemical (IHC) staining was performed using the monoclonal antibodies Em2G11, specific for *E. multilocularis*, and Eg2 (reactive with both species) (antibodies provided by Prof. Deplazes, University of Zurich). No specific immunoreactivity was observed with either antibody, indicating a negative result for both species.

Genomic DNA was extracted from FFPE tissue and used for PCR amplification of the mitochondrial markers *cox1*, *nad1*, and *12S*. Expected amplicons were obtained for *nad1* and *12S*, while *cox1* required a nested PCR approach (Table 1). Sequencing and maximum‑likelihood phylogenetic analysis confirmed the parasite’s identity (Additional file 1: Fig. S1), with BLAST results showing over 99% similarity to *T. martis* homologous sequences*.*

The sequences obtained were deposited in GenBank under accession numbers PV933839, PV933844, and PV941853.

During the follow-up, the patient was interviewed by the healthcare personnel using a structured questionnaire to investigate potential routes of exposure. The patient, a retired schoolteacher, had spent her entire life the same province, with the exception of two trips to Australia and Sri Lanka. She resided in an urban area where she maintained a vegetable garden and regularly consumed eggs purchased from a local producer. She also owned a second property in a nearby mountainous area, where she spent time engaging in recreational activities such as hiking. She reported no contact with pets, rodents, or mustelids, and denied eating berries while hiking. However, she did report drinking spring water during these excursions.

**Table 1:** Primers used for PCR and sequencing

Primer name	Sequence (5′–3′)	PCR fragment (bp)	Gene	Reference
F/COI	TTGAATTTGCCACGTTTGAATGC	1175	*cox1*	[30]
R/COI	GAACCTAACGACATAACATAATGA			
EGCOI1	TTTTTTGGTCATCCAGAAGTTTAT	444		[31]
EGCOI2	TAAAGAAAGAACATAATGAAAATG			
JB11	GATTCGTAAGGGGCCTAATA	528	*nad1*	[32]
JB12	CCACTAACTAATTCACTTTC			
P60	TTAAGATATATGTGGTACAGGATTAGATACCC	376	*12 s*	[33]
P375	AACCGAGGGTGACGGGCGGTGTGTACC			

## Discussion

Human infection with *T. martis* is exceptionally rare: the present case represents the eighth documented to date, all reported since 2010 and geographically clustered in western/central Europe [5–11] (Additional file 2: Fig. S1).

Clinical manifestations depend on the location of the cyst: cerebral cases, including the present, presented with headache, motor deficits, and neurological symptoms; peritoneal cases presented with ascites and abdominal pain, while ocular infections were characterized by visual alterations. In all cases, including ours, surgical removal of the lesion was performed, followed by molecular diagnosis. Ultimately, in some cases, treatment with albendazole, sometimes combined with praziquantel and corticosteroids, was administered (Table 2). When investigated, no secondary lesions were found elsewhere in the body. When follow-up was recorded, complete symptom remission was reported. By contrast, *T. martis* infection appears more severe in nonhuman primates, with several fatal cases in lemurs and only one successfully treated macaque [15, 16].

*Taenia martis* has been reported in the fauna of Europe and North America, yet human cases remain confined to Europe. In North America, *T. martis* (formerly *T. martis americana* [12]) has been reported in mustelids; however, it has not been recorded in wildlife since the 1990s, except for a single recent case in a Canadian fisher [17], and no human infections have ever been reported. It must be pointed out that reviewing the presence of *T. martis americana* may be challenged by the use of alternative names in literature, such as *Taenia sibirica* and *Taenia* cf *intermedia* [18, 19].

In Europe, *T. martis* has been detected in animal hosts from Ireland to southern Italy, including Lithuania, Belarus, and Serbia [20–24]. Recently, it has also been identified in several exotic mammals under human care in the Czech Republic [25].

Human cases concentrate in Germany and neighboring regions, despite the broader distribution of the parasite and its animal hosts.

Despite the recognition of *T. martis* human infection, which has prompted the implementation of dedicated molecular tests [26], serology still lacks specific assays. Over the seven cases documented previously, serological tests have been applied in five cases with ambivalent results. Only enzyme-linked immunosorbent assay (ELISA) for *T. solium*, in a 24-year-old woman with peritoneal *T. martis* cysticercosis, gave a clear positive result [9], while western blot and indirect fluorescent antibody test (IFAT) for *T. solium* (both in cerebral cases) and ELISA for *E. granulosus* (in one case of peritoneal cysticercosis) returned weak positivity [8–10]. In the case documented by Eggink et al., the use of recombinant antigen immunoblot (rT24H antigen and LLGP) tested negative [11].

In the present case, the western blot for *Taenia solium* was unequivocally negative. Regarding the histological diagnosis, in the absence of distinctive morphological features, histology alone would not have been sufficient to support a diagnosis of NCC in the absence of distinctive morphological features and molecular analysis. Nevertheless, tubular structures of approximately 15 μm in diameter were observed within the necrotic core. Similar elements have been reported in a case of *Versteria rafei* infection, with authors suggesting that these structures might correspond to larval stages of *Versteria* [27]. We cannot exclude that such findings represent larval stages of taeniid parasites in general.

Molecular testing therefore represents the most accurate diagnostic tool, as demonstrated in the present case, which was confirmed through three PCR targets and phylogenetic analysis (Additional file 2: Fig. S1); nevertheless, the availability of antigen-based confirmatory assays would provide an important complementary resource.

In summary, this case highlights the critical contribution of molecular approaches to the diagnosis of NCC caused by less common *Taenia* species, particularly when considering the following points: (a) routine serology of cysticercosis does not include specific assays, which may lead to misdiagnosis, especially since clinical symptoms are not specific; (b) histopathology can be highly indicative but requires the expertise of trained personnel, and the presence of distinctive morphological features (e.g., hooks, rostellum) is not always guaranteed, as in the present case; (c) it provides valuable insights into the epidemiology of these infections, which are difficult to trace due to their long incubation period.

Interestingly, most reported cases of *T. martis* cysticercosis, both past and present cases, have occurred in women (age range 24–72 years), with only one case reported in a young boy [11]. Although biological susceptibility has been hypothesized [1], the pattern could equally reflect chance, reporting bias, or gender-related differences in exposure. Across cases, several common habits or circumstances were noted, such as residing near or spending time in forested areas. Frequently, the patients also reported gardening and growing their own vegetables (5,6,7,9,10) [5, 7–10]. In three cases, the patients reported seeing martens around their homes [7, 9, 10]. In the Dutch case [11], a survey of a stone marten population in a neighboring province revealed that 15% of animals were molecularly positive for *T. martis*, and the sequences obtained from these martens, as well as from a squirrel, were 100% identical to those of the young patient. Notably, no contact with wild fauna was ever reported, and pets, when present, were excluded as potential carriers, supporting the possibility of environmental exposure rather than direct animal contact. In our case, the patient had traveled to Australia and in Sri Lanka in 2011 and 2019, respectively. The epidemiology of *T. martis* excludes these localities as source sites of infections. Alternatively, the patient’s reported activities—such as gardening, hiking, and drinking spring water near her household in the province of Bergamo—though unconfirmed, may represent potential routes of exposure.

Pine and stone martens, the main hosts of *T. martis*, are widely distributed across Italy, inhabiting mostly forested areas, with the stone marten also thriving near human environments. The two martens are sympatric across almost the entire Italian territory, with few exceptions; however, in the Italian Alps, the stone marten is likely predominant [28]. Infections by *T. martis* have been detected in martens from both northern and southern regions, as well as in bank voles (*Clethrionomys glareolus*) [29], and a fatal case occurred in a lemur in Rome [15]. Given this host distribution and the scattered reports of infection, the parasite is likely present throughout much of the Italian peninsula.

The reporting of such cases must reach clinicians, ensuring that, when the clinical presentation is typical of cysticercosis, negative serology does not lead to the exclusion of cestode infection from differential diagnosis, thereby preventing appropriate treatment. The development of species‑specific or broader‑range immunological assays targeting *Taenia* species other than *T. solium* would therefore be valuable, although their feasibility is inevitably limited by the rarity of these infections. Secondly, it should also contribute, within a One Health approach, to properly educating the public, especially those who, as current and past cases suggest, are exposed to environmental or occupational wildlife-related risk factors.

**Table 2:** Summary of therapeutic treatments in the reviewed cases and the present case

Case (gender, age)	Location of the lesion	Therapeutic treatment	Reference
Woman, 43 years old	Ocular	Prior to surgery: albendazole (400 mg, twice daily); dexamethasone (20 mg, daily)	[5]
Woman, 44 years old	Cerebral	Post-surgery: praziquantel (50 mg/kg, daily) for 15 days; albendazole (15 mg/kg, daily) for 1 month combined with corticosteroids (1 mg/kg, daily)	[6]
Woman, 70 years old	Ocular	Post-surgery: albendazole (400 mg, twice daily) for 7 days	[7]
Woman, 36 years old	Peritoneal	Post-surgery: albendazole (400 mg, twice daily) for 4 weeks	[8]
Woman, 24 years old	Peritoneal	Treatment offered, patient refused	[9]
Woman, 55 years old	Cerebral	Prior to surgery: dexamethasone. Post-surgery: albendazole and praziquantel as for *T. solium* treatment	[10]
Boy, 11 years old	Cerebral	Post-surgery: albendazole (twice daily) for 7 days	[11]
Woman, 72 years old	Cerebral	Prior to and post-surgery: corticosteroids	Present case

## Supplementary Information

Additional file 1: Text S1. Phylogenetic relationships based on the cox1 (322 bp) and nad1 (B) (458 bp) sequence alignments of *T. martis* obtained in this study (black diamond), along with selected reference sequences from GenBank representing *T. martis* and other related cestodes. The phylogenetic trees were constructed using the maximum likelihood method in CLC Genomics Workbench, displaying only branches supported by bootstrap values >60% (1000 replicates). Reference sequences are annotated with GenBank accession numbers, scientific names, and country of origin for *T. martis*. The scale bar indicates the number of nucleotide substitutions per site. Text S1. Phylogenetic relationships based on the cox1 (322 bp) and nad1 (B) (458 bp) sequence alignments of *T. martis* obtained in this study (black diamond), along with selected reference sequences from GenBank representing *T. martis* and other related cestodes. The phylogenetic trees were constructed using the maximum likelihood method in CLC Genomics Workbench, displaying only branches supported by bootstrap values >60% (1000 replicates). Reference sequences are annotated with GenBank accession numbers, scientific names, and country of origin for *T. martis*. The scale bar indicates the number of nucleotide substitutions per site.

Additional file 2: Text S1. Map of Europe showing reported human cases of *T. martis* cysticercosis. Cases are annotated by anatomical localization, year of diagnosis, patient age and sex, and literature reference. Different symbols indicate the patient’s geographical origin or the clinical referral center.

## Data Availability

All data generated or analyzed during this case report are included in this published article or are available as supplementary data. Sequence data were submitted to GenBank (accession numbers: PV933839, PV933844, and PV941853).
