# Supplementary figures and images for: Human neurocysticercosis unexpectedly caused by Taenia martis in Italy: a case report and literature review
Source: Parasit Vectors. 2026 May 15;19:273. doi: 10.1186/s13071-026-07420-2 (PMC13330242; doi:10.1186/s13071-026-07420-2)

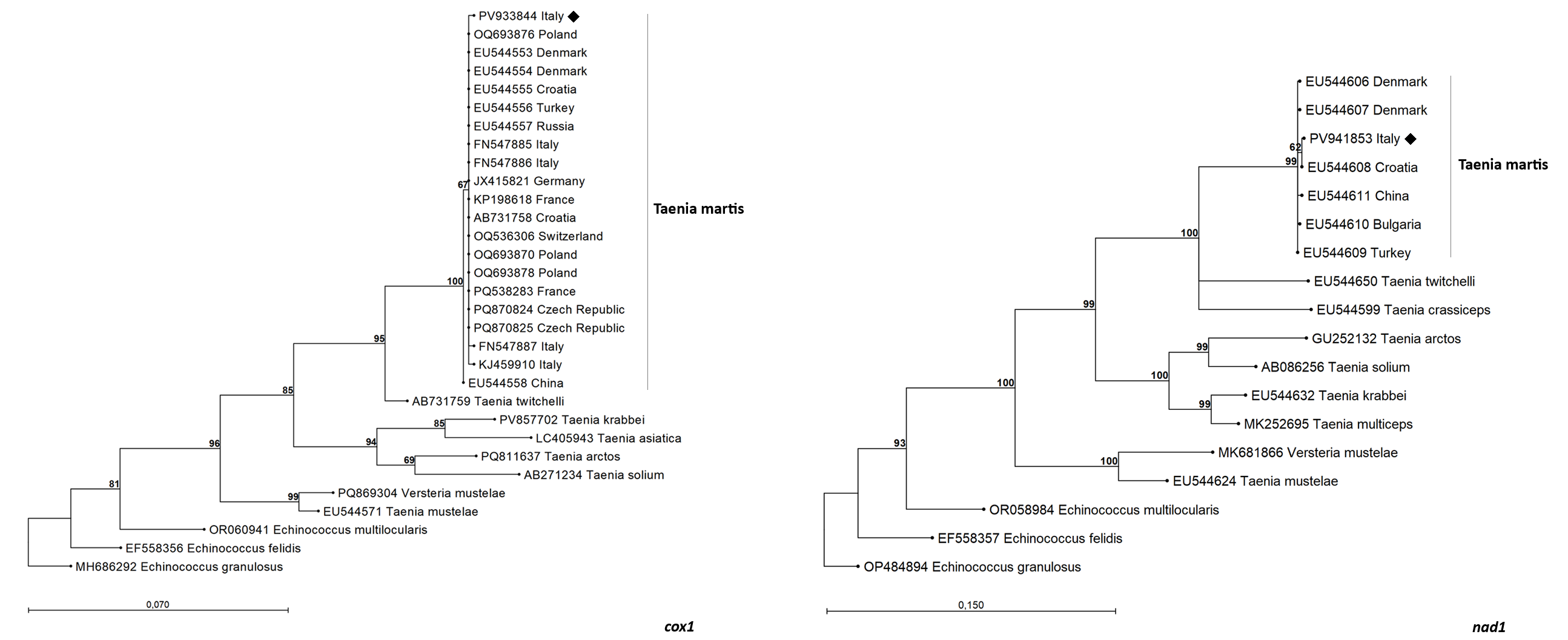

Supplement: Supplementary file 1 — Additional file 1: Text S1. Phylogenetic relationships based on the cox1 (322 bp) and nad1 (B) (458 bp) sequence alignments of T. martis obtained in this study (black diamond), along with selected reference sequences from GenBank representing T. martis and other related cestodes. The phylogenetic trees were constructed using the maximum likelihood method in CLC Genomics Workbench, displaying only branches supported by bootstrap values >60% (1000 replicates). Reference sequences are annotated with GenBank accession numbers, scientific names, and country of origin for T. martis. The scale bar indicates the number of nucleotide substitutions per site. Text S1. Phylogenetic relationships based on the cox1 (322 bp) and nad1 (B) (458 bp) sequence alignments of T. martis obtained in this study (black diamond), along with selected reference sequences from GenBank representing T. martis and other related cestodes. The phylogenetic trees were constructed using the maximum likelihood method in CLC Genomics Workbench, displaying only branches supported by bootstrap values >60% (1000 replicates). Reference sequences are annotated with GenBank accession numbers, scientific names, and country of origin for T. martis. The scale bar indicates the number of nucleotide substitutions per site. [file 13071_2026_7420_MOESM1_ESM.tif]

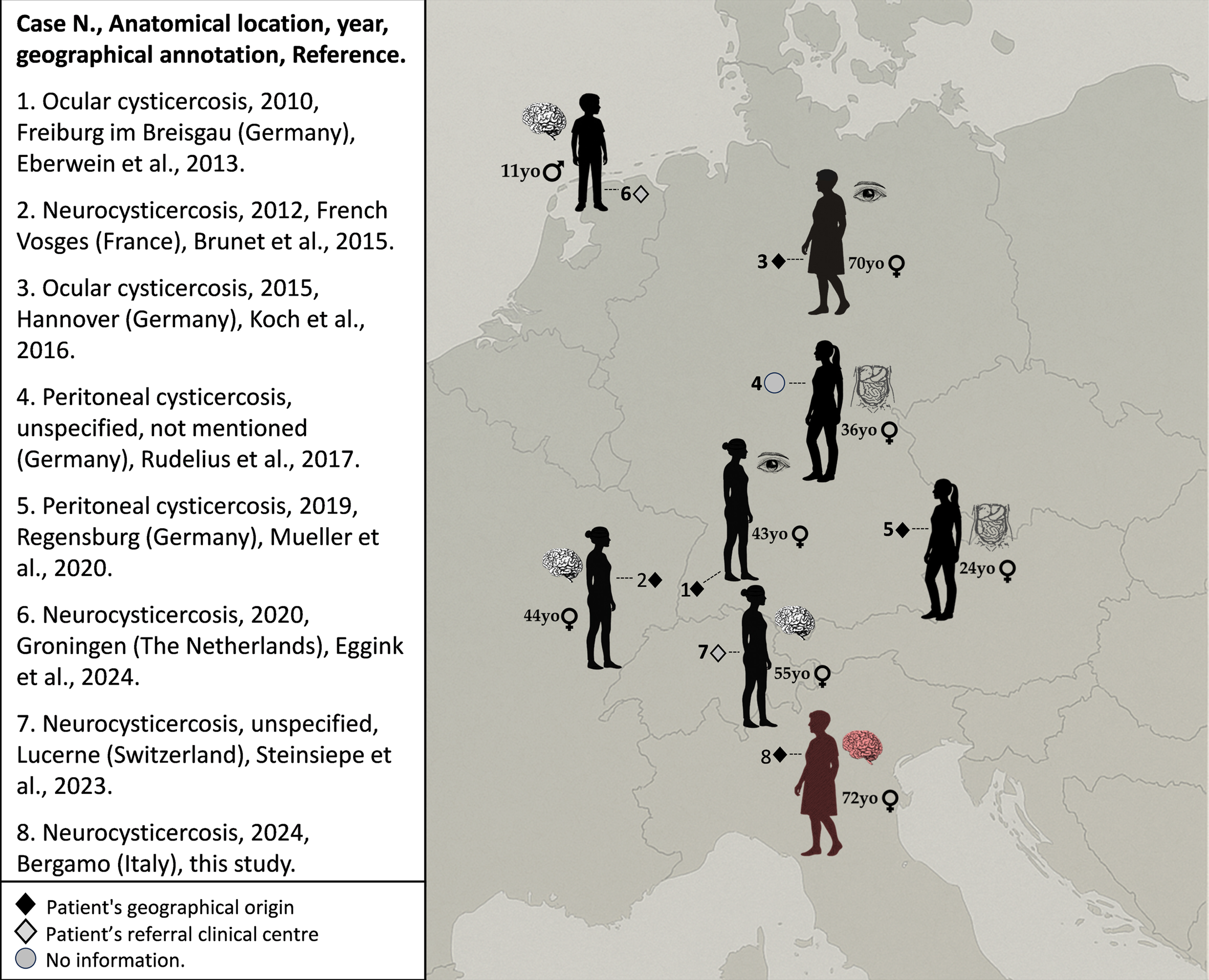

Supplement: Supplementary file 2 — Additional file 2: Text S1. Map of Europe showing reported human cases of T. martis cysticercosis. Cases are annotated by anatomical localization, year of diagnosis, patient age and sex, and literature reference. Different symbols indicate the patient’s geographical origin or the clinical referral center. [file 13071_2026_7420_MOESM2_ESM.tif]
